# Breast cancer PAM50 signature: correlation and concordance between RNA-Seq and digital multiplexed gene expression technologies in a triple negative breast cancer series

**DOI:** 10.1186/s12864-019-5849-0

**Published:** 2019-06-03

**Authors:** A. C. Picornell, I. Echavarria, E. Alvarez, S. López-Tarruella, Y. Jerez, K. Hoadley, J. S. Parker, M. del Monte-Millán, R. Ramos-Medina, J. Gayarre, I. Ocaña, M. Cebollero, T. Massarrah, F. Moreno, J. A. García Saenz, H. Gómez Moreno, A. Ballesteros, M. Ruiz Borrego, C. M. Perou, M. Martin

**Affiliations:** 1 Instituto de Investigación Sanitaria Gregorio Marañón (IiSGM), Doctor Esquerdo 46, 28007 Madrid, Spain; 2 Hospital General Universitario Gregorio Marañón, Madrid, Spain; 3 Medical Oncology Service, Instituto de Investigación Sanitaria Gregorio Marañón (IiSGM). CiberOnc, Hospital General Universitario Gregorio Marañón, Madrid, Spain; 4 Department of Genetics, University of North Carolina at Chapel Hill, Chapel Hill, NC USA; 5 Anatomical Pathology Service, Hospital General Universitario Gregorio Marañón, Madrid, Spain; 6 Medical Oncology Service, Hospital Universitario Clínico San Carlos, Madrid, Spain; 7 Medicina Oncológic, Instituto Nacional de Enfermedades Neoplásicas (INEN), Lima, Peru; 8 Medical Oncology Service, Hospital Universitario de La Princesa, Madrid, Spain; 9 Hospital Virgen del Rocío, Sevilla, Spain; 10 Department of Genetics, Lineberger Comprehensive Cancer Center, University of North Carolina, Chapel Hill, NC USA; 11 Hospital General Universitario Gregorio Marañón, Instituto de Investigación Sanitaria Gregorio Marañón (IiSGM), Universidad Complutense, CiberOnc, GEICAM, Madrid, Spain

**Keywords:** PAM50, Breast cancer, Triple negative breast cancer, RNA-Seq, Multiplexed gene expression

## Abstract

**Background:**

Full RNA-Seq is a fundamental research tool for whole transcriptome analysis. However, it is too costly and time consuming to be used in routine clinical practice. We evaluated the transcript quantification agreement between RNA-Seq and a digital multiplexed gene expression platform, and the subtype call after running the PAM50 assay in a series of breast cancer patients classified as triple negative by IHC/FISH. The goal of this study is to analyze the concordance between both expression platforms overall, and for calling PAM50 triple negative breast cancer intrinsic subtypes in particular.

**Results:**

The analyses were performed in paraffin-embedded tissues from 96 patients recruited in a multicenter, prospective, non-randomized neoadjuvant triple negative breast cancer trial (NCT01560663). Pre-treatment core biopsies were obtained following clinical practice guidelines and conserved as FFPE for further RNA extraction. PAM50 was performed on both digital multiplexed gene expression and RNA-Seq platforms. Subtype assignment was based on the nearest centroid classification following this procedure for both platforms and it was concordant on 96% of the cases (*N* = 96). In four cases, digital multiplexed gene expression analysis and RNA-Seq were discordant. The Spearman correlation to each of the centroids and the risk of recurrence were above 0.89 in both platforms while the agreement on Proliferation Score reached up to 0.97. In addition, 82% of the individual PAM50 genes showed a correlation coefficient > 0.80.

**Conclusions:**

In our analysis, the subtype calling in most of the samples was concordant in both platforms and the potential discordances had reduced clinical implications in terms of prognosis. If speed and cost are the main driving forces then the preferred technique is the digital multiplexed platform, while if whole genome patterns and subtype are the driving forces, then RNA-Seq is the preferred method.

**Electronic supplementary material:**

The online version of this article (10.1186/s12864-019-5849-0) contains supplementary material, which is available to authorized users.

## Background

Gene expression signatures are becoming a key tool for decision-making in oncology, and especially in breast cancer. In 2000, Perou et al. identified 4 intrinsic subtypes of breast cancer with clinical implications from microarray gene expression data: Luminal A (LumA), Luminal B (LumB), HER2-enriched and Basal-like [1–3]. These breast cancer subtypes yielded a superior prognostic impact than classical immunohistochemistry (IHC) factors. Almost a decade later Parker et al. developed from the initial intrinsic subtypes, a 50-gene signature for subtype assignment [4].

Initially developed on microarray data, PAM50 is being successfully used in digital multiplexed gene expression platforms such as NanoString nCounter®, which is the basis for the Prosigna® test. The latter includes the PAM50 assay in combination with a clinical factor (i.e. tumor size) and has been approved for the risk of distant relapse estimation in postmenopausal women with hormone receptor-positive, node negative or node positive early stage breast cancer patients; and is a daily-used tool assessing the indication of adjuvant chemotherapy [5, 6].

The NanoString nCounter® system enables gene expression analysis through direct multiplexed measurements. This technology is based on 2 probes specific to each gene of interest, a capture probe and a reporter probe, consisting of a complementary sequence to the target messenger RNA (mRNA) coupled to a color-coded tag [7]. Unique pairs of capture and reporter probes are designed for each gene of interest, and up to 800 genes can be analyzed simultaneously for a single sample. Tumor RNA and probes are hybridized together and following purification and alignment, they are identified and quantified by the analyzer. NanoString has proved to be highly reproducible, and has shown a high concordance between fresh-frozen (FF) and formalin-fixed paraffin-embedded (FFPE) derived RNAs [8].

On the other hand, RNA-Seq has become the cornerstone of modern whole transcriptome analyses. It represents a useful tool for discovery and validation of biomarkers. The use of FFPE has been a concern in the past but several studies observed that this kind of samples are suitable to be used in RNA-Seq platforms assessing for gene expression analyses, and comparable to fresh frozen tissue [9]. From the technical point of view, typical RNA-Seq protocols based on poly(A) enrichment of the mRNA in order to remove ribosomal RNA, fail to capture the partially degraded mRNA in FFPE samples. However this limitation can be overcome by using Ribo-Zero-Seq and it has been proved that it performs as good as microarrays or RNA-Seq based on poly(A) enrichment [10]. However, its processing time requirements and economic costs make it difficult its implementation in daily clinical practice scenario. In this study, we compared the performance of the intrinsic subtype determination by PAM50 along with the risk of recurrence (ROR) estimation from both platforms: RNA-Seq and NanoString nCounter®, by using the same samples on both and directly comparing results.

## Results

### Sample quality

Overall, 96 samples were successfully processed and had sufficient RNA for both NanoString nCounter® and RNA-Seq transcript quantification. The mean RNA concentration from the FFPE samples was 146.9 ng/μl, mean RNA integrity number (RIN) value was 2.015 (min/max: 1.1/3.7; 95% CI: 1.899–2.130) and its mean A260/A280 ratio was 1.98 (min/max: 1.83/2.06; 95% CI: 1.971–1.979) (Additional file 2: Table S2, online only). None of the samples used in RNA-Seq had measurable amounts of rRNA and all the samples presented optimal metrics. Moreover, the none of the samples processed in NanoString nCounter® presented technical issues and just three of them presented negligible control/count hints. Both quality control (QC) reports are in the respective Additional files 4 and 5 (online only).

### Intrinsic subtype calling

The intrinsic subtype calling results in both RNA-Seq and NanoString nCounter® are shown in the Additional file 1: Table S1 (online only).

**Fig. 1:**
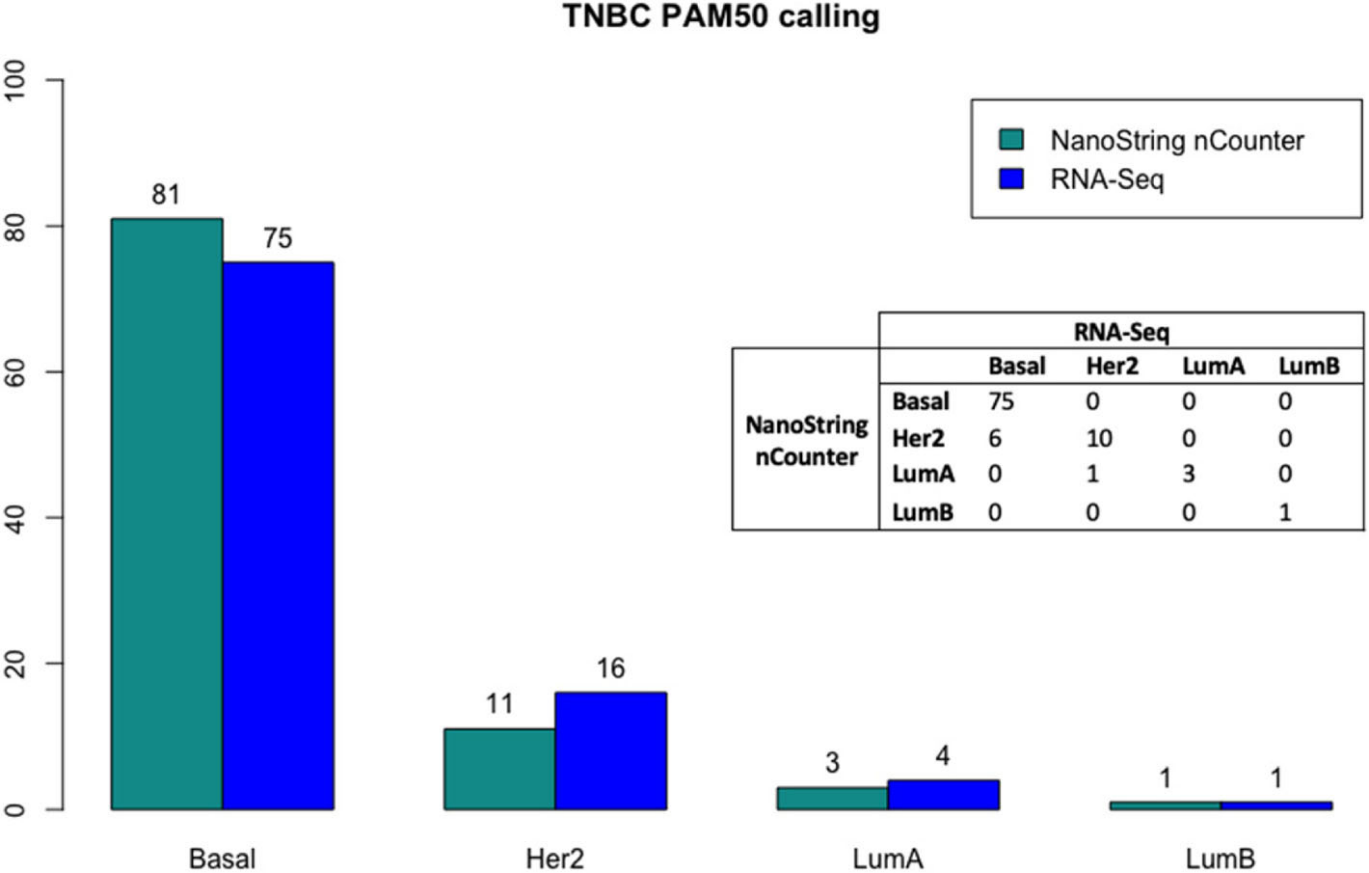
PAM50 subtype calls by technique. Barplot represents counts of samples per subtype and technique. The cross table shows in detail the discordances between both platforms

As displayed in Fig. 1, NanoString nCounter® classified 84.3% of the patients as Basal-like, 11.5% as HER2-enriched, 3.1% as LumA and 1.0% as LumB. RNA-Seq intrinsic subtype distribution was as follows: 78.1% basal-like, 16.7% HER2-enriched, 4.2% LumA, 1.0% LumB.

As displayed in Table 1, we had 7 patients with discordant subtype calls by the two techniques (7.3%). However, we observed that 3 patients had their second closest centroids within a distance ≤0.10 (range: 0.01 to 0.10), one of them concordant with the call offered by the other technique. The remaining 4 discordant cases showed real discordances in their calls and centroids proximity. Taking this information into account, we considered that subtype calling agreed on 96% of the cases (NanoString nCounter®/RNA-Seq discordances: 3 Basal-like/HER2-enriched and 1 HER2-enriched/LumA). We reevaluated the discordant samples in the PAM50 assay output. We only observed that one sample (HUGM-0022) had a low confidence score (0.42) in RNA-Seq due to extremely similar centroid correlation values, thus we really cannot classify it with a high degree of confidence.

**Table 1:**
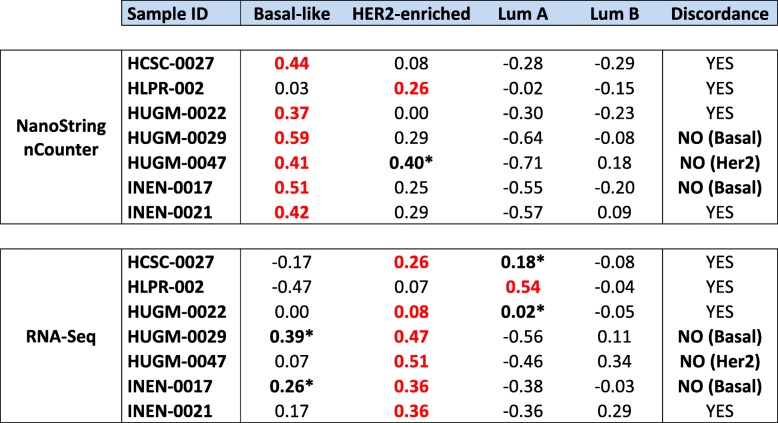
Centroid correlation for the potential discordant sample calls

These measures are extracted from the PAM50 assay outcome (Additional file 1: Table S1). The sample’s subtype classification is assigned to the centroid with the highest correlation (in bold red). When the second centroid has a value close to the highest one (difference less or equal to 0.1) the classification is ambiguous being possible any of both subtypes (bold *). The *Discordance* column summarizes whether a real discordance is observed in a sample or just a scenario where two centroid correlations are almost equivalent (HUGM-0047 in NanoString nCounter® is a paradigmatic case)

### PAM50 centroids and risk of recurrence

**Fig. 2:**
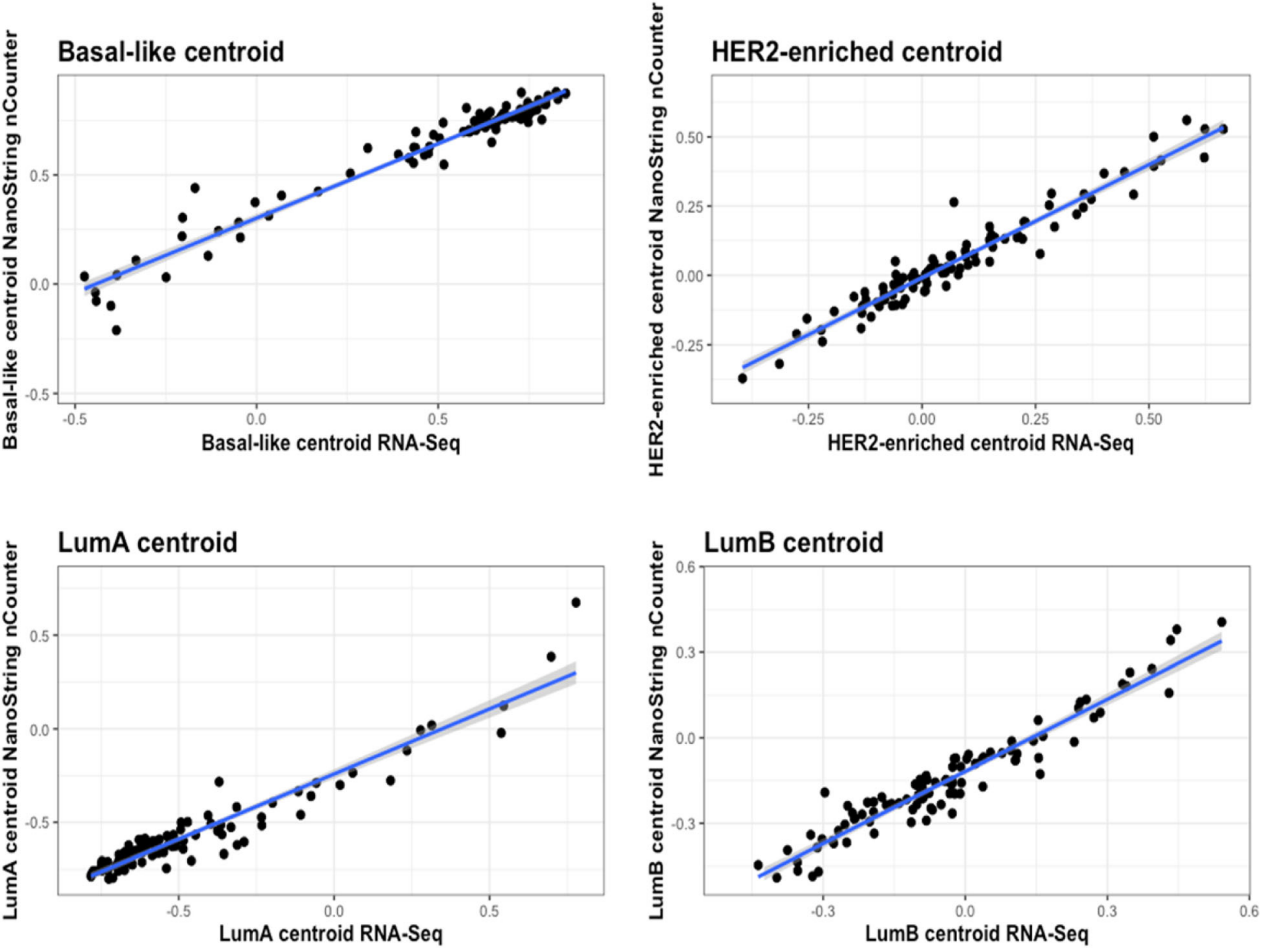
Separate centroid correlation when NanoString nCounter® and RNA-Seq platforms are compared. The blue line represents the linear regression. The grey area surrounding it represents the confidence interval

We next analyzed the correlation to each of the centroids obtained through NanoString nCounter® and RNA-Seq data, and we observed that the Spearman’s rho was above 0.95 for all the centroids (Basal-like/HER2-enriched 0.97, LumA 0.95, and LumB 0.96) (Fig. 2).

**Fig. 3:**
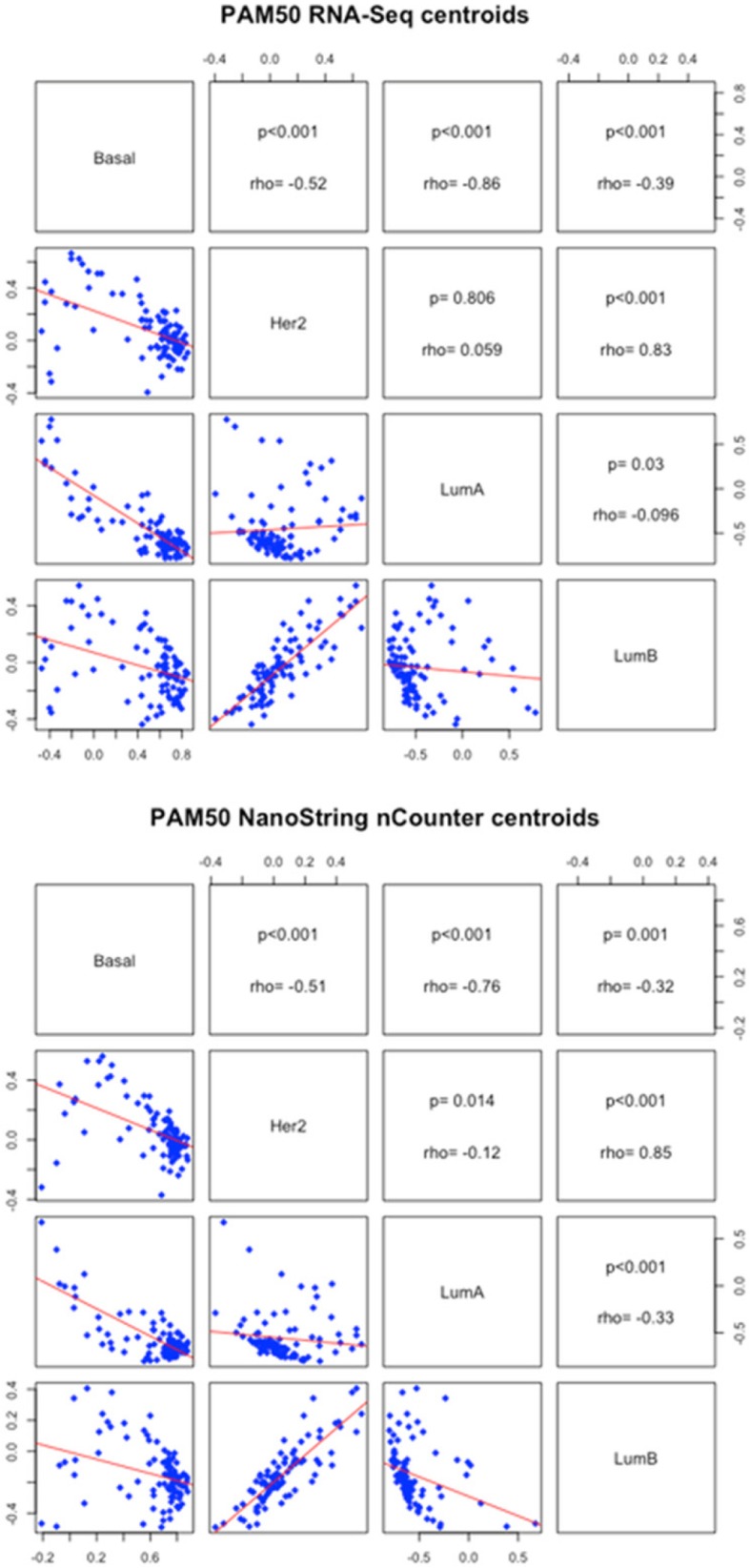
Correlation of the correlation to the centroids in both platforms obtained in the PAM50 subtype classifier

In addition, we evaluated the correlation between each of the different centroids for both platforms and we observed similar results. The highest positive correlation was for the HER2-enriched and LumB centroids, with a Spearman’s rho of 0.83 and 0.85 (*p* < 0.01) with RNA-Seq and NanoString nCounter®, respectively. On the other hand, Basal-like and LumA centroids had the strongest inverse correlation (rho 0.86 and 0.76, *p* < 0.01 with RNA-Seq and NanoString nCounter®, respectively) (Fig. 3).

**Fig. 4:**
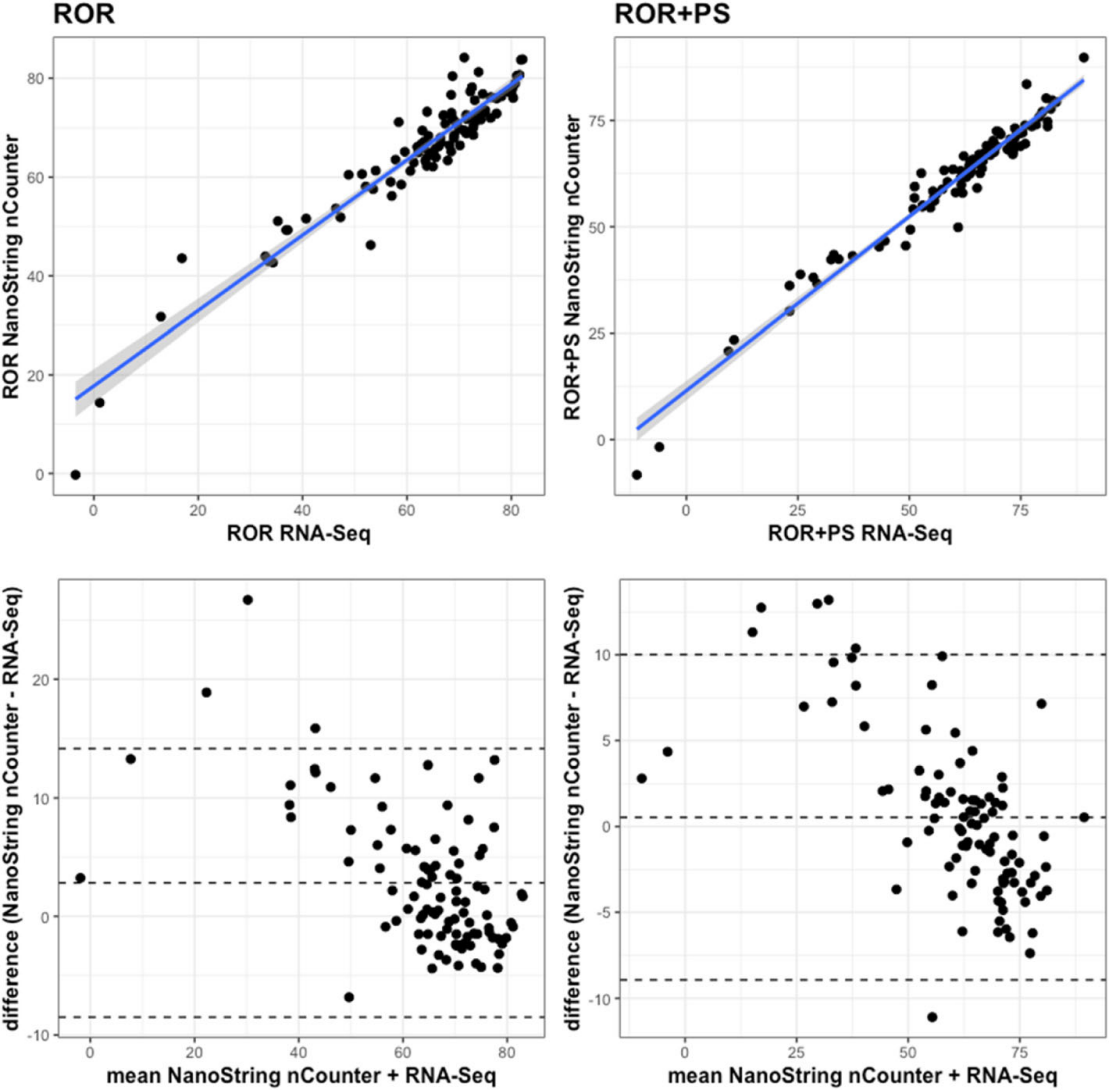
Correlation of ROR and ROR + PS and their associated Bland-Altman plots in both platforms. The upper/lower dashed lines in the Bland-Altman plots represent the mean difference +/− 1.96 * standard deviation. The central dashed line represents the mean difference

The risk of recurrence score (ROR), and considering the role of the Proliferation Score (ROR + PS), had a Spearman’s rho of 0.90 and 0.97, respectively. Thus, in terms of ROR, the results show an extremely high correlated scenario. We observed high agreement between techniques in the Bland-Altman plots displayed in Fig. 4, as most of the differences remain close to the null baseline level within the confidence interval. In addition, the intraclass correlation coefficient (ICC) for ROR reached 0.93 [0.89–0.95] and ROR + PS reached 0.96 [0.94–0.97].

Additional measures such as expression level of HER2, along with the Proliferation Score, also showed a high degree of correlation between both platforms with a Spearman’s rho 0.96 and 0.97, respectively.

### Individual gene correlation

**Fig. 5:**
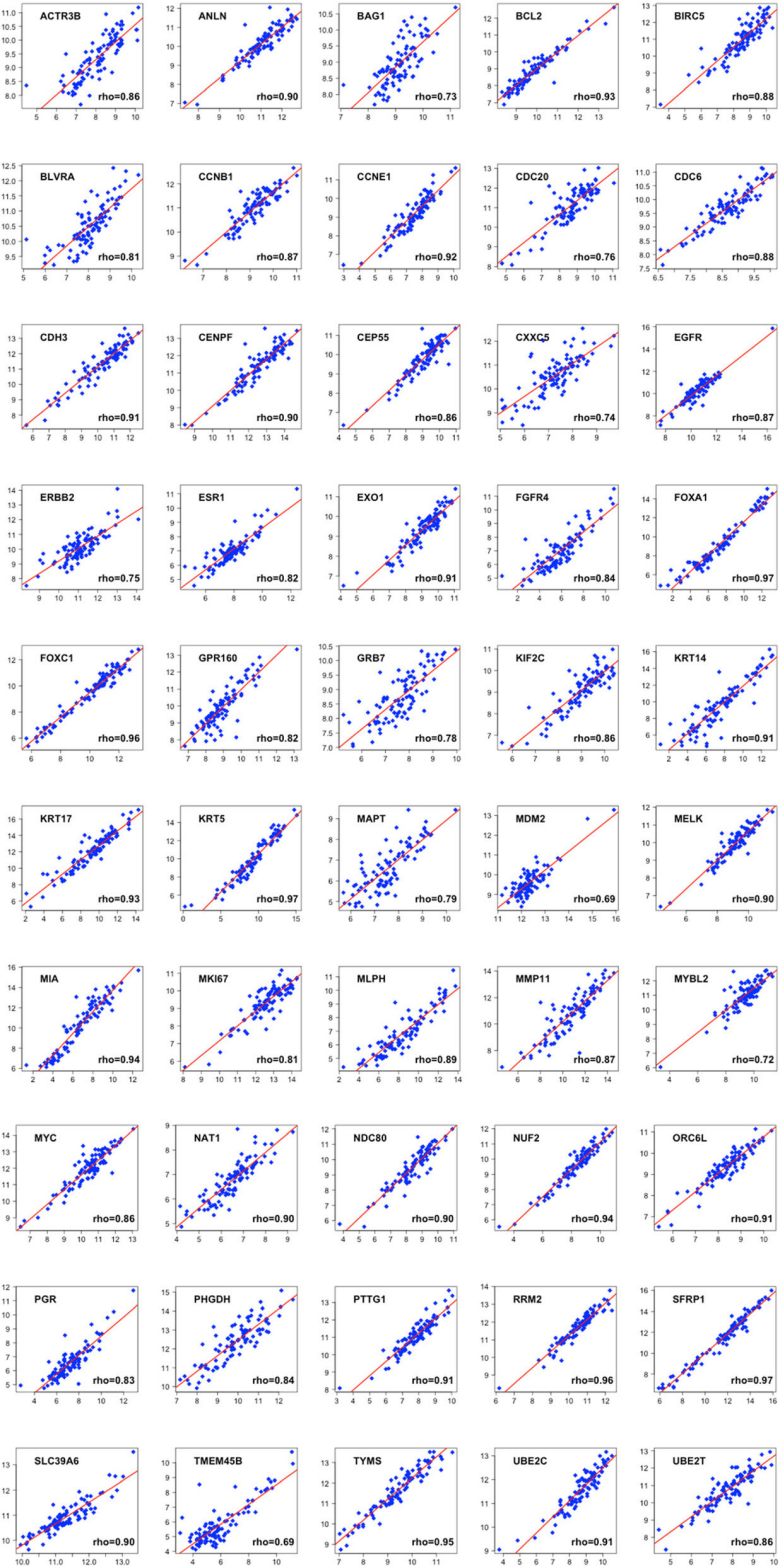
Normalized gene expression levels for each gene contained in the PAM50 assay. The log2 normalized counts for RNA-Seq are represented in the X-axis and those for NanoString nCounter® are represented in the Y-axis. The red line represents the LOWESS smoother, which uses locally weighted polynomial regression

We lastly evaluated the correlation coefficients for each of the 50 genes in the PAM50 gene list. We measured the expression levels in log2 scale in both platforms. We observed that in our dataset 23 genes had a correlation greater than 0.9, 18 genes between 0.8 and 0.9, 7 genes between 0.7 and 0.8 and only 2 genes had a correlation lower than 0.7. The median ICC was 0.90 (mean = 0.88) (Fig. 5 and Additional file 3: Table S3, online only).

## Discussion

The goal of the study was assessing the reproducibility of PAM50 intrinsic subtype when using RNA-Seq and NanoString nCounter® data from FFPE tissue obtained from a triple negative breast cancer (TNBC) patient cohort. We noticed that the PAM50 subtype calling was concordant on 96% of the cases and the expression in genes that comprise the PAM50 assay had a median ICC of 0.90.

PAM50 was originally developed and validated using microarray data from 1753 genes, but since then it has been transferred into a wide variety of platforms. Interestingly, PAM50 performance has been evaluated by comparing quantitative real-time reverse-transcription-PCR (qRT-PCR) and NanoString nCounter® [11]. That study obtained an overall concordance of 0.94 in subtype calls, 0.98 for ROR and 0.95 for ROR + PS. Regarding individual gene expression, median ICC was 0.90 [11]. These measures are very similar to ours comparing NanoString nCounter® and RNA-Seq, as we presented in the Results Section.

In this TNBC cohort 4 samples out of 96 were misclassified in the subtype calling. While this might be concerning from the patient care perspective, it is strongly suggested in these cases to evaluate the ROR and ROR + PS, because from the clinical point of view the ROR-score group is more important to select therapy (chemotherapy vs no chemotherapy) than the plain subtype calling. The PAM50 assay provides numeric and categorical values for both scores and we observed in the misclassified samples the assigned risk group remained the same except in one patient with discordant low/medium ROR (Table 2).

**Table 2:** Risk of Recurrence (ROR) for the potential discordant sample calls

	Sample ID	ROR	ROR-Group	ROR-PS	ROR + PS-Group	Discordance
NanoString nCounter	HCSC-0027	51.10	med	43.42	med	NO
	HLPR-002	43.57	med	23.44	med	YES
	HUGM-0022	51.60	med	42.28	med	NO
	HUGM-0029	73.51	high	68.98	high	NO
	HUGM-0047	81.24	high	72.48	high	NO
	INEN-0017	66.66	high	55.051	high	NO
	INEN-0021	72.50	high	65.40	high	NO
RNA-Seq	HCSC-0027	35.25	med	33.04	med	NO
	HLPR-002	16.87	low	10.68	low	YES
	HUGM-0022	40.68	med	32.44	med	NO
	HUGM-0029	75.02	high	69.59	high	NO
	HUGM-0047	73.73	high	69.58	high	NO
	INEN-0017	62.68	high	52.98	high	NO
	INEN-0021	66.98	high	64.52	high	NO

These measures are extracted from the PAM50 assay outcome (Additional file 1: Table S1). Two measures regarding the risk of recurrence are reported: ROR, which takes into account only the subtype calling; and ROR + PS, which considers also the Proliferation Score. The latter is defined as the mean expression level for the proliferation genes: *CCNB1, UBE2C, BIRC5, KNTC2, CDC20, PTTG1, RRM2, MKI67, TYMS, CEP55* and *CDCA1*. The ROR(+PS)-Group columns gives a categorical classification in terms of risk: high, medium (med) and low

Perou, Sørlie, Hu, Nielsen et al. evaluated the prognostic effect of PAM50 genes using the qRT-PCR from FFPE samples, and demonstrated its superiority to standard clinicopathological factors in predicting long-term survival of estrogen receptor positive tumors [12, 13]. There is significant evidence that IHC is not a reliable surrogate of genomic intrinsic subtype, and that gene expression methods have a higher predictive and prognostic value than IHC [12, 14, 15]. Moreover, in a comprehensive review in breast cancer gene-expression based assays by Prat et al. it is shown that the concordance between two different ER/PR testing methods based on IHC falls below the highest levels of reproducibility/concordance expected in daily clinical use [16].

The kind of samples to be processed is often a major factor in deciding which technology should be used to quantify transcripts and perform the PAM50 assay. In medical research the FFPE are the most common sources of archived material because they are cheap, easy to process and stable for a very long time. The PAM50 PCR-based classifier has been validated and translated into the NanoString nCounter® platform, because it previously demonstrated higher performance than PCR for FFPE data [8]. Since this platform does not require an amplification step, it enables a more sensitive analysis of degraded mRNA from FFPE samples [17, 18]. Although it seems that NanoString and DNA microarrays show a good correlation, similar to the one found when comparing distinct microarrays platforms [7], correlations between FF microarrays and FFPE was moderate due to RNA poor quality in the FFPE samples. According to Chen et al. NanoString detected a higher number of transcripts than microarrays (88.4% vs. 82.6%) [17].

Several studies for both oncologic and non-oncologic diseases have shown a good correlation between gene expression data from RNA-Seq and NanoString nCounter® (*R*^2^ = 0.90 for FFPE samples in idiopathic pulmonary fibrosis) [19]. Particularly in breast cancer, NanoString nCounter® and RNA-Seq using Illumina TruSeq Ribo-Zero-Gold RNA-Seq enable reliable gene expression analysis from degraded FFPE RNA. This study encourages the role of NanoString nCounter® as a validation platform from data discovered by RNA-Seq, with a high reproducibility of both techniques (*R*^2^ = 0.99 for technical replicates), high correlation with FF matched samples (*R*^2^ = 0.874 for NanoString) and a high correlation between both platforms (*R*^2^ = 0.838) [8].

While RNA-Seq technologies can be used in multiple research scenarios [20] a digital multiplexed platform provides several advantages over RNA-Seq technologies in daily clinical practice in terms of cost, amount of needed RNA, computational cost and the use of FFPE samples. This last factor is often crucial in clinical research as fresh frozen (FF) tissue is generally unavailable due to the infrastructure and timing required, making it difficult to reach the necessary number of samples in multicenter studies. However, that means increased sequencing costs in order to get enough reads to achieve adequate coverage of coding genes. In this project the estimated full cost for processing and sequencing an RNA-Seq sample was around 800–1000 USD; on the other hand, the cost of processing a sample in NanoString nCounter® was just around 190 USD. The overall financial burden to perform PAM50 using NanoString nCounter® in a diagnostic laboratory is lower than any RNA-Seq option in terms of equipment and experts, as the latter may require outsourcing the analyses. This situation usually entails longer delivery times as long as the sample processing and transcript quantification using NanoString nCounter® can be achieved in less than 28 h. In addition, the amount of RNA needed in both platforms is substantially different: in our case we ran the analyses using 500–1000 ng in RNA-Seq and only 250 ng in NanoString nCounter®; the latter claims to keep its performance with only 125 ng. Additionally, the computational costs can be several orders of magnitude higher in processing RNA-Seq than NanoString nCounter® samples because the former needs computationally demanding processes of alignment and transcript quantification.

Finally, our study shows that PAM50 intrinsic subtype in TNBC patients, which are mostly basal-like, is reproducible between different platforms. This encourages the solidity of the classification. Moreover, our comparison comes from the same FFPE-extracted RNA, directly comparing the platform with no RNA quality bias.

## Conclusions

The PAM50 subtype calling agreement between RNA-Seq and NanoString nCounter® transcript quantification technologies was evaluated in FFPE tissue samples obtained from a TNBC patient cohort. We observed that the subtype call was concordant in most of the samples and the potential discordances had reduced clinical implications in terms of prognosis. Although PAM50 can be used with RNA-Seq data and it shows similar results to NanoString nCounter®; the former is still difficult to use in daily clinical practice due to its processing time requirements and economic costs, unlike the discussed digital multiplexed option.

## Methods

### Patients and samples

The analyses were performed in FFPE samples from 96 patients recruited in a previously described multicenter, prospective, non-randomized neoadjuvant TNBC trial (NCT01560663) [21]. This cohort consisted of early stage TNBC patients, defined as estrogen and progesterone receptor (ER and PR) < 1% and HER2-negative according to the ASCO-CAP guidelines, candidate for neoadjuvant treatment with carboplatin and docetaxel [22, 23].

This trial included patients from 7 centers across Spain and Peru. Pre-treatment core biopsies were performed and conserved as FFPE following each institution protocol for further RNA extraction.

This study was reviewed and approved by the Ethical Board at all the participating institutions, and all patients included were required to sign a written informed consent. All clinical data and samples were anonymized.

### RNA extraction

FFPE blocks were centralized and included in a biologic sample bank at the Translational Oncology Laboratory (LAOT), which belongs to the Medical Oncology Department at the Hospital General Universitario Gregorio Marañón, registered at the Instituto de Salud Carlos III; in Madrid, Spain.

RNA extraction and PAM50 intrinsic subtyping on the nCounter® platform were performed at the LAOT. Invasive tumor from haematoxilin and eosin stained slides was delimited by a pathologist at the LAOT and was subsequently microdissected in 10 μm slides. The number of slides recommended based on the measured tumor surface area on the hematoxylin and eosin (H&E) stained slide; 4-19 mm^2^: 6 unstained 10 μm slides; 20–99 mm^2^: 3 unstained 10 μm slides; ≥100 mm^2^: 1 unstained 10 μm slide. RNA was then extracted using the RNeasy FFPE Kit (Qiagen), which is specially designed for purification of total RNA from FFPE tissue sections by isolating RNA molecules longer than 70 nucleotides. Firstly, all paraffin is removed from freshly cut FFPE tissue sections by treating with deparaffinization solution or using an alternative deparaffinization method. Next, samples are incubated in an optimized lysis buffer, which contains proteinase K, to release RNA from the sections. A short incubation at a higher temperature partially reverses formalin crosslinking of the released nucleic acids, improving RNA yield and quality, as well as RNA performance in downstream enzymatic assays. This is followed by DNase treatment that is optimized to eliminate all genomic DNA, including very small DNA fragments that are often present in FFPE samples after prolonged formalin fixation and/or long storage times. Next, the lysate is mixed with Buffer RBC. Ethanol is added to provide appropriate binding conditions for RNA, and the sample is then applied to a RNeasy MinElute spin column, where the total RNA binds to the membrane and contaminants are efficiently washed away. RNA is then eluted in a minimum of 14 μl of RNase-free water. RNA concentration and quality were assessed with Nanodrop 2000 Spectophotometer (Thermo Scientific NanoDrop Products) according to A260/280 ratio. RIN was evaluated with the TapeStation 2200 (Agilent Technologies, Germany).

### NanoString nCounter®

From an initial panel of 110 genes, we analyzed the expression of the 50 genes included in the PAM50 assay and 5 additional housekeeper genes described by Parker et al [4]. In the raw nCounter® transcript quantification the background was corrected using the negative probes and normalized with their mean minus 2 standard deviations, and those values were normalized by calculating the geometric mean of the 5 housekeeper genes. Subtype classification was assigned based on the nearest of the 5 centroids.

### RNA-Seq

Sequencing was performed at the University of North Carolina (UNC) at Chapel Hill (NC, US). 300-1000 ng of total FFPE RNA was used to create an RNA-Seq library using the Illumina TruSeq Ribo-Zero Gold Kit (RS - 1 22–2301 or RS - 122 - 2302). Libraries were then sequenced 2 per lane on a HiSeq2500 with 48x7x48 bp configuration. Alignment against GRCh37 and transcript quantification was done using MapSplice [23] v2.2.1 and RSEM [4] v1.3.0 using the UCSC GAF2.1 KnownGenes using UBU v1.0 (https://github.com/mozack/ubu), respectively. Samples were normalized to a fixed upper quartile (1000 genes) followed by log2 transformation. The PAM50 algorithm was run according to the scripts provided by Parker et al [4] adjusting the genes to a previously determined estrogen receptor balanced median from FFPE samples assayed by TruSeq Ribo-Zero Gold to adjust samples to match the training set. The code and median adjustment value are provided as Additional file 6.

### Quality control analysis

The quality control for the RNA-Seq samples was assessed using the .*fastq* files in FastQC v0.11.8 [24]. We also evaluated the potential presence of ribosomal RNA sequences obtained from the UCSC Genome Browser [25] using FastQ Screen v0.13.0 [26]. The QC for the NanoString nCounter® samples was done using NanoStringQCPro [27].

### Statistical analysis

We used R [28] v3.3.3 in order to evaluate the transcript quantification in both platforms, and the PAM50 subtype calling along with its associated centroid values and the ROR. All comparisons between continuous variables were performed using the Spearman correlation. The inter-rater agreement analysis for molecular subtype classification was based on the Cohen’s kappa calculation. For continuous variables ICC two way mixed effect single measures [29] was calculated. Given ROR importance in diagnostic, a Bland-Altman plot was constructed to further assess agreement between platforms.

## Additional files

Additional file 1: **Table S1.** PAM50 output for RNA-Seq and NanoString nCounter. PAM50 table of results for RNA-Seq and NanoString nCounter®. It is reported for each sample and platform: the centroid correlation, the subtype call with its confidence, the ROR and ROR + PS values and groups, the Proliferation Score; and the ER/HER2 gene expression. (XLSX 90 kb)

Additional file 2: **Table S2.** RNA Sample Quality Control. The RNA concentration measured in each sample, absorbance values for A260 and A280, the A260/A280 ratio and the measured RIN. Descriptive statistics are provided for A260/A280 ratio and RIN. (XLSX 16 kb)

Additional file 3: **Table S3.** PAM50 genes correlation and inter-rate agreement analyses. Correlation analysis using the Spearman’s rho where the gene expression measured in RNA-Seq and NanoString nCounter® are compared. The inter-rater agreement analysis for the PAM50 genes was based on the Cohen’s kappa calculation using two-way mixed effect single measures. The statistics and *p*-values are provided in both analyses. (XLSX 17 kb)

Additional file 4: QC – Fastq. RNA-Seq data Quality Control. FastQC and FastQ Screen reports summarized using MultiQC. All the information is summarized in an interactive .html. Additionally, a table is provided to associate the sample IDs mentioned in the manuscript with the IDs generated during the sequencing process. (ZIP 5962 kb)

Additional file 5: QC – NanoString. NanoString nCounter data Quality Control. NanoStringQCPro reports in .html files. Technical, control and count-based metrics are reported. Additionally, a table is provided to associate the sample IDs mentioned in the manuscript with the IDs generated during the NanoString nCounter® quantification process. (ZIP 15743 kb)

Additional file 6: Code. Normalization and PAM50 scripts for both platforms. Scripts used to perform the PAM50 assay in RNA-Seq and NanoString nCounter® platforms, along with additional technique-specific and transcript ID files. (ZIP 91 kb)

## Data Availability

The datasets used and/or analysed during the current study are available from the corresponding author on reasonable request.

## Abbreviations

ER: Estrogen receptor
FF: Fresh-frozen
FFPE: Formalin-fixed paraffin-embedded
FISH: Fluorescence in situ hybridization
HER2: Human epidermal growth factor receptor 2
ICC: Intraclass correlation coefficient
IHC: Immunohistochemistry
mRNA: messenger RNA
PAM50: Prediction analysis for microarrays (50)
PCR: Polymerase chain reaction
PR: Progesterone receptor
PS: Proliferation Score
QC: Quality control
qRT-PCR: Real-Time Quantitative Reverse Transcription PCR
RIN: RNA integrity number
RNA: Ribonucleic acid
RNA-Seq: RNA sequencing
ROR: Risk of recurrence
TNBC: Triple negative breast cancer
UCSC: University of California, Santa Cruz
