# Supplementary material for: Breast cancer PAM50 signature: correlation and concordance between RNA-Seq and digital multiplexed gene expression technologies in a triple negative breast cancer series
Source: BMC Genomics. 2019 Jun 3;20:452. doi: 10.1186/s12864-019-5849-0 (PMC6547580; doi:10.1186/s12864-019-5849-0)
Supplement: Supplementary file 5 — QC – NanoString. NanoString nCounter data Quality Control. NanoStringQCPro reports in .html files. Technical, control and count-based metrics are reported. Additionally, a table is provided to associate the sample IDs mentioned in the manuscript with the IDs generated during the NanoString nCounter® quantification process. (ZIP 15743 kb) [file 12864_2019_5849_MOESM5_ESM.zip › qc-nanostring/nanostringqcpro_report/LAOT-TNBC-20150630-qc.html]

Data set summary


Table of Contents

- Data set summary
- Technical metrics
  - Fields of view
  - Binding density
  - Technical metrics summary
- Control probe metrics
  - Negative and positive control probes
  - Sample-specific background level
  - Spike-in linearity
  - Control probe interquartile range
  - Positive control scaling factors
  - Control probe metrics summary
- Count-based metrics
  - Total counts and positive control counts
  - Gene detection count
  - Count-based metrics summary
- Flagged samples summary
- Similarity between samples
- Probe level QC
  - Probe behavior in blank samples
  - Probes with extreme counts
- QC metrics in normalized data
  - Housekeeping genes
  - Housekeeping and global median normalization
- Recommendations
  - QC flag summary
  - Clustering and QC-flagged samples
- SessionInfo

# Data set summary

The data set was composed of 12 samples and 110 genes.

# Technical metrics

As a first step in quality control, we assess whether technical aspects differed across samples.

## Fields of view

According to the nCounter® Expression Data Analysis Guide, “the nCounter® Digital Analyzer images each lane in discrete units, called fields of view (FOV). Optical issues, such as an inability to focus due to bubbles or insufficient oiling of the cartridge, can prevent successful imaging of a FOV. The Digital Analyzer reports the number of FOVs successfully imaged as 'FOV Counted'. Significant discrepancy between the number of FOV for which imaging was attempted (FOV Count) and for which imaging was successful (FOV Counted) may be indicative of an issue with imaging performance.” Samples with less than 80% FOV Counted are highlighted in red:

## Binding density

As the nCounter® Digital Analyzer only counts non-overlapping barcodes, significant data loss could occur if there are too many barcodes in the imaged area. The nCounter® Digital Analyzer calculates Binding Density as a measure of image saturation. For details, please see the nCounter® Expression Data Analysis Guide. According to NanoString®, Binding Density which is either too low (< 0.05) or too high (> 2.25) is indicative of technical issues (e.g., excess RNA input); such samples are marked in red:

## Technical metrics summary

Assessment of these technical factors resulted in flagging of 0 sample(s).

# Control probe metrics

## Negative and positive control probes

All NanoString® runs contain negative control probes that are designed to not hybridize to any human target. The background signal reported by the negative control probes typically differs somewhat from probe to probe.

Positive control probes and associated spike-ins are also part of every NanoString® run. For positive controls, there should be a linear relationship between the nominal input concentration and the reported counts. Because spike-ins differ by a common dilution factor, this linearity should be visible in the positive control box plot when counts are plotted on a logarithmic scale:

## Sample-specific background level

Negative control probes can be used to determine lane-specific (i.e., sample-specific) background noise levels (to see the full plot, click on the preview image below):

## Spike-in linearity

The slope estimate produced by fitting the linear model *log(counts) ~ log(input)* shows how well an increase in spike-in input is reflected by an increase in counts. Thresholds for identification of outliers (dotted red lines) were determined using the *cutoffByMMAD* method (see package documentation for this function); outliers, if any, are colored in red:

## Control probe interquartile range

We can make use of the negative and positive controls for further quality control. For instance, an increased or decreased spread of the data stemming from the negative or positive controls might be indicative of technical issues (such as increased background or decreased dynamic range). Below, we plot the interquartile range (IQR) for negative and positive control probes. Thresholds for identification of outliers (dotted red lines) were determined using the *cutoffByMMAD* method. Outliers, if any, are colored in red:

## Positive control scaling factors

Finally, the overall signal for the spike-in controls may identify lanes experiencing technical issues. NanoString® suggests calculating a positive control scaling factor; if this scaling factor is outside a range of 0.3 - 3, it may indicate significant under-performance, and such results should be interpreted with caution. Samples with positive control scaling factors outside the recommended range are colored in red; for more details, please see the vignette as well as the help page for the *posCtrlNorm* function.

## Control probe metrics summary

0 sample(s) were flagged based on the performance of the controls.

# Count-based metrics

Problematic samples may exhibit unusual overall counts, or have many targets with counts below the background estimate.

In each plot in this section, blank samples (if present in the experiment) are displayed as triangles. Thresholds for identification of outliers (dotted red lines) were determined using the *cutoffByMMAD* method (see package documentation for this function); outliers, if any, are colored in red.

## Total counts and positive control counts

We first plot the sum of all counts for each sample, excluding control probes. These counts reflect the amount of RNA input in each sample; any outliers might not have had enough input to permit reliable detection of genes with low expression levels. The next plot shows the ratio of positive control counts to all non-control counts for each sample.

## Gene detection count

For each sample, we plot the number of genes with counts that were below the estimated background, defining the LLOD using *cutoffByMMAD*. Samples with more than 20 % of genes missing are colored in red:

## Count-based metrics summary

Assessment of these metrics resulted in flagging of 1 sample(s).

# Flagged samples summary

In total, 1 sample(s) were flagged based on quality metrics.

# Similarity between samples

Below, sample to sample correlations are clustered (distfun = “euclidean”, hclustfun = “complete”) and visualized in a heatmap. Deeper color represents a stronger correlation (to see the full-size image, click on the preview image below):

(omitted since heatmaps were not enabled)

# Probe level QC

## Probe behavior in blank samples

Blank samples allow background signal estimation in a probe-specific fashion. Blank samples also enable detection of other suspicious behavior, e.g., elevated background counts that might indicate problems during probe-target hybridization. If blank samples were included in the data set, you will see a boxplot showing each gene's counts in the blanks as well as red dots showing the estimated probe-specific background (to see the full plot, click on the preview image below):

(omitted since no blank samples were found in the input)

## Probes with extreme counts

Probes which rarely produced signals above the LLOD are not informative for your experiment. On the other hand, if a small number of probes scavenge the majority of counts because their targets were extremely highly expressed, detection of low abundance transcripts may be compromised.

To address these two issues, we first look at the least detectable genes. If multiple sample types were defined, we determine for each sample type separately the genes that produced counts above the estimated background in less than 50% of these samples. (Note: up to 20 least detectable genes are shown for each sample type.)

Next, we show the five genes that produced the highest average counts; again, if multiple sample types were defined, the data will be split accordingly:

# QC metrics in normalized data

## Housekeeping genes

After other preprocessing steps, data need to be normalized to correct for count differences caused by varying RNA input amounts. A popular method for doing so is using gene expression levels of housekeeping genes. We first inspect the expression of housekeeping genes across all samples to assess their suitability as normalization factors:

Additional metrics for assessing housekeeping gene behavior are available here.

## Housekeeping and global median normalization

Another way to normalize data is to use information of all genes measured in the panel and to normalize by their median. Here we show the overall distribution of counts in all samples using raw data, and data normalized by housekeeping genes (if applicable) and scaling to global median:

Then, we have a closer look at how different normalization methods perform with respect to inferred gene-gene correlation. If you have prior knowledge about which genes you expect to cluster together, the heatmaps allow you to check the impact of normalization.

Global normalization (to see the full-size image, click on the preview image below):Housekeeping normalization (to see the full-size image, click on the preview image below):

(omitted since heatmaps were not enabled)

# Recommendations

## QC flag summary

The following table lists all samples and marks those flagged by QC metrics with an “X”:

| SampleIdentifier | SampleName | TechnicalFlags | ControlFlags | CountFlags | SampleType |
| --- | --- | --- | --- | --- | --- |
| 20150630\_20150629-1\_11B0015859\_01.RCC | 11B0015859 |  |  |  | TNBC |
| 20150630\_20150629-1\_11B22197\_08.RCC | 11B22197 |  |  |  | TNBC |
| 20150630\_20150629-1\_13B40318\_06.RCC | 13B40318 |  |  |  | TNBC |
| 20150630\_20150629-1\_14-100177\_02.RCC | 14-100177 |  |  |  | TNBC |
| 20150630\_20150629-1\_14-101736\_03.RCC | 14-101736 |  |  |  | TNBC |
| 20150630\_20150629-1\_14-102050\_04.RCC | 14-102050 |  |  |  | TNBC |
| 20150630\_20150629-1\_14B0002164\_11.RCC | 14B0002164 |  |  |  | TNBC |
| 20150630\_20150629-1\_14B0002337\_10.RCC | 14B0002337 |  |  |  | TNBC |
| 20150630\_20150629-1\_14B0009265A1\_09.RCC | 14B0009265A1 |  |  |  | TNBC |
| 20150630\_20150629-1\_14B13442A2\_07.RCC | 14B13442A2 |  |  |  | TNBC |
| 20150630\_20150629-1\_14B807\_05.RCC | 14B807 |  |  |  | TNBC |
| 20150630\_20150629-1\_43768\_12.RCC | 43768 |  |  | X | TNBC |

Download as tab-delimited text file

## Clustering and QC-flagged samples

Below, we center each gene across all samples and perform a clustering based on correlation. Blue means lower expression than the mean of all samples, red higher expression. The colored bars above the heatmap indicate samples flagged in different QC categories (to see the full-size image, click on the preview image below):

(omitted since heatmaps were not enabled)

Finally, we exclude all flagged samples and recluster. The colored bar above the plot now represents the total number of counts per sample, with deeper color reflecting more counts (to see the full-size image, click on the preview image below):

(omitted since heatmaps were not enabled)

# SessionInfo

This report was created by root on 2018-06-11 11:53:15.

R version 3.4.3 (2017-11-30)
Platform: x86\_64-pc-linux-gnu (64-bit)
Running under: Debian GNU/Linux 9 (stretch)
Matrix products: default
BLAS: /usr/lib/openblas-base/libblas.so.3
LAPACK: /usr/lib/libopenblasp-r0.2.19.so
locale:
[1] LC\_CTYPE=en\_US.UTF-8 LC\_NUMERIC=C LC\_TIME=en\_US.UTF-8
[4] LC\_COLLATE=en\_US.UTF-8 LC\_MONETARY=en\_US.UTF-8 LC\_MESSAGES=C
[7] LC\_PAPER=en\_US.UTF-8 LC\_NAME=C LC\_ADDRESS=C
[10] LC\_TELEPHONE=C LC\_MEASUREMENT=en\_US.UTF-8 LC\_IDENTIFICATION=C
attached base packages:
[1] parallel stats4 methods stats graphics grDevices utils datasets
[9] base
other attached packages:
[1] NanoStringQCPro\_1.10.0 org.Hs.eg.db\_3.5.0 AnnotationDbi\_1.40.0
[4] IRanges\_2.12.0 S4Vectors\_0.16.0 Biobase\_2.38.0
[7] BiocGenerics\_0.24.0 BiocInstaller\_1.28.0
loaded via a namespace (and not attached):
[1] Rcpp\_0.12.15 highr\_0.6 pillar\_1.2.1 compiler\_3.4.3
[5] RColorBrewer\_1.1-2 plyr\_1.8.4 rngtools\_1.2.4 iterators\_1.0.9
[9] tools\_3.4.3 digest\_0.6.15 bit\_1.1-12 evaluate\_0.10.1
[13] RSQLite\_2.0 memoise\_1.1.0 tibble\_1.4.2 gtable\_0.2.0
[17] gridBase\_0.4-7 NMF\_0.21.0 png\_0.1-7 pkgconfig\_2.0.1
[21] rlang\_0.2.0 foreach\_1.4.4 registry\_0.5 DBI\_0.8
[25] stringr\_1.3.0 pkgmaker\_0.22 cluster\_2.0.6 knitr\_1.20
[29] bit64\_0.9-7 grid\_3.4.3 reshape2\_1.4.3 magrittr\_1.5
[33] ggplot2\_2.2.1 blob\_1.1.0 scales\_0.5.0 codetools\_0.2-15
[37] xtable\_1.8-2 colorspace\_1.3-2 stringi\_1.1.7 lazyeval\_0.2.1
[41] doParallel\_1.0.11 munsell\_0.4.3
